# The *P* element invaded rapidly and caused hybrid dysgenesis in natural populations of *Drosophila simulans* in Japan

**DOI:** 10.1002/ece3.4239

**Published:** 2018-09-04

**Authors:** Yusaku Yoshitake, Nobuyuki Inomata, Mai Sano, Yasuko Kato, Masanobu Itoh

**Affiliations:** ^1^ Department of Applied Biology Kyoto Institute of Technology Kyoto Japan; ^2^ Department of Environmental Science International College of Arts and Sciences Fukuoka Women's University Fukuoka Japan; ^3^ Center for Advanced Insect Research Promotion (CAIRP) Kyoto Institute of Technology Kyoto Japan

**Keywords:** *Drosophila*, horizontal transmission, hybrid dysgenesis, *P* element, transposon

## Abstract

Transposable elements not only can change genomic positions and disperse across the gene pool, but also can jump to another species through horizontal transmission. Of late, the *P* element, a DNA transposon in insects, was shown to cross the genetic boundary from *Drosophila melanogaster* to *D. simulans* in Europe around 2006. To understand the dynamics of transposable elements, especially in the early stages of invasion, we examined 63 lines of *D. simulans* from 11 natural populations in Japan established in 1976–2015. Based on PCR analyses, *P* elements were demonstrated to exist in Japan in 2008 and later. One copy of the full‐length *P* element was identified and mapped to a site on chromosome 3 L in a genome. All of 18 copies of *P* elements examined shared “A” at the nucleotide position 2040, which is representative of the direct descendants of the original *P* element that invaded in *D. simulans*. We also found that some lines having *P* elements can induce intensive gonadal dysgenesis in *D. simulans* at 29°C. Our present results imply that *P* elements in *D. simulans* arrived at the east end of Asia just a few years later than or almost simultaneously to the initial invasion in Europe, Africa, and North America, suggesting a more astonishingly rapid spread than previously assumed.

## 1 INTRODUCTION

Transposable elements (TEs) are one of the general components of genomes, especially in eukaryotes (Bergman, Quesneville, Anxolabéhère, & Ashburner, 2006; Craig, Craigie, Gellert, & Lambowitz, 2002; Kidwell & Lisch, 1997). TEs change genomic position, replicate their own copies in a genome, and, thus, can spread all across a gene pool via the usual inheritance. TEs also leap occasionally from one species to another through horizontal transmission (HT), by which TEs can move between organisms by means other than ordinary vertical gene transmission from parents to offspring (Kidwell, 1993; Le Rouzic & Capy, 2005). Molecular mechanisms of HT are almost unknown, but most probable molecular vehicles are bacteria and viruses that can shuttle TEs between organisms with ecological connections, for instance, prey–predator and host–parasite interactions (Drezen et al., 2017; Schaack, Gilbert, & Feschotte, 2010; Venner et al., 2017), although a mite, *Proctolaelaps regalis*, was proposed as a vector of the HT in *Drosophila* (Houck, Clark, Peterson, & Kidwell, 1991). The evolutionary relationship between TEs and the host genome remains controversial. TEs play important roles in genetic diversity of the hosts by insertion/deletion, illegitimate recombination, and domestication. However, their propagating behavior appear to have a selfish unconcern about the fitness of the hosts (Biemont & Vieira, 2006; Feschotte & Pritham, 2007; Hua‐Van, Le Rouzic, Boutin, Filee, & Capy, 2011; Orgel & Crick, 1980). For a successful invasion, TE needs a suitable cellular environment. Colonized once in a new gene pool, TEs can increase in copy number despite deleterious consequences for the host. The host, on the other hand, quickly develops regulatory systems for suppressing TEs’ transposition under a permissible level (Lee & Langley, 2012; Lewis et al., 2018; Senti, Jurczak, Sachidanandam, & Brennecke, 2015). As a consequence of such arm races, invaded TEs would spread first, but be totally inactive for a long time, and only their remnant sequences would remain. For bypassing this extinction scenario, another HT is the only escape route, except for domestication (Le Rouzic & Capy, 2009; Pinsker, Haring, Hagemann, & Miller, 2001; Schaack et al., 2010).

The *P* element, a DNA transposon in insects, is one of the most popular model systems of TEs (O'Hare & Rubin, 1983) and the causative factor of P‐M hybrid dysgenesis in *Drosophila melanogaster* (Bingham, Kidwell, & Rubin, 1982). A P strain carries many copies of *P* elements in genome, and an M strain has no copy. In the germline of F1 progeny of a cross between M females and P males, *P* elements transpose and lead to hybrid dysgenesis, including symptoms such as gonadal dysgenesis (GD) sterility, elevated mutations, segregation distortion, and male recombination (Ashburner, Golic, & Hawley, 2005; Kidwell, 1994). *P* elements recently invaded *D. melanogaster* by HT from another *Drosophila* species, probably *D. willistoni,* in the middle of the 20th century (Daniels, Peterson, Strausbaugh, Kidwell, & Chovnick, 1990; Kidwell, 1979). *P* elements dispersed worldwide and are currently virtually ubiquitous in wild populations of *D. melanogaster* (Anxolabéhère, Kidwell, & Periquet, 1988; Anxolabéhère, Nouaud, Periquet, & Tchen, 1985; Bonnivard & Higuet, 1999), Therefore, true M strains are limited to old laboratory lines that were established before the invasion of the *P* element (Kidwell, Kidwell, & Sved, 1977).

**Figure 1:**
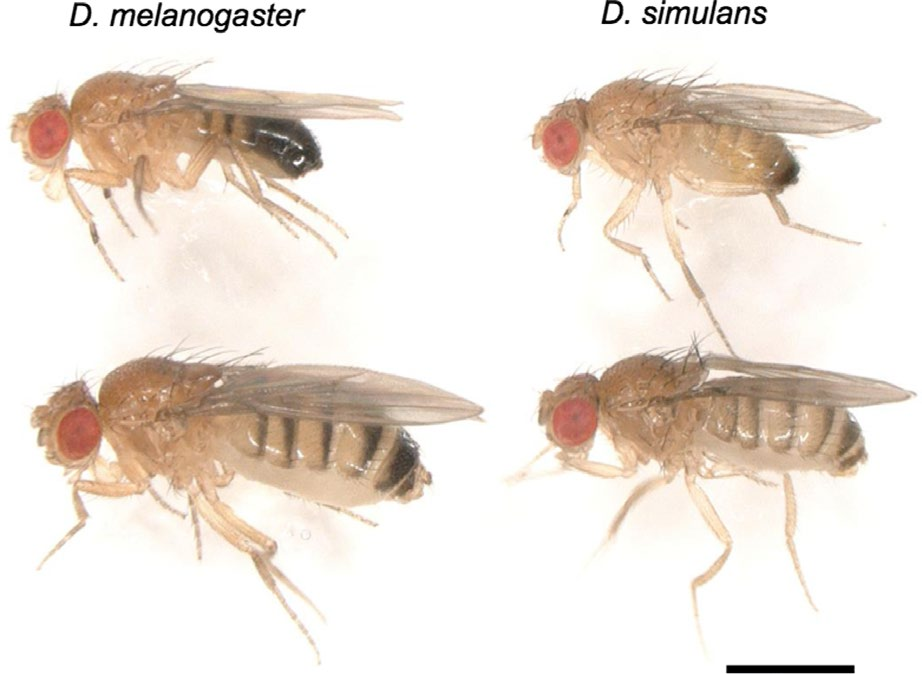
A picture of *Drosophila melanogaster* (left) and *D. simulans* (right). Males (upper) and females (lower). The photograph was taken with a digital microscope VHX‐6000 (KEYENCE, Japan). Bar: 1 mm

The fruit flies *D. simulans* and *D. melanogaster* (Figure 1) are closely related in the *Drosophila melanogaster* species subgroup of the genus *Drosophila*. They are highly similar in morphology, ecologically, and genetically (reviewed by Capy, Gibert, & Boussy, (2004)). *P* elements were known to invade only *D. melanogaster*, but not *D. simulans* (Brookfield, 1991). This was rather enigmatic, because the *P* element was repeatedly shown to be active in *D. simulans* if artificially introduced (Daniels, Strausbaugh, & Armstrong, 1985; Montchamp‐Moreau, 1990; Scavarda & Hartl, 1984) and able to increase in number with structural decay with time as in *D. melanogaster* (Daniels, Chovnick, & Kidwell, 1989; Higuet, Merçot, Allouis, & Montchamp‐Moreau, 1996; Kimura & Kidwell, 1994; Scavarda & Hartl, 1987). However, an occurrence of HT, of *P* elements transferring from *D. melanogaster* to *D. simulans*, was recently reported by Kofler, Hill, Nolte, Betancourt, & Schlötterer (2015), who demonstrated that isofemale lines of *D. simulans* established in North America in 2010 and South Africa in 2012 carried many *P* elements in their genomes. They indicated that some of *P* elements of *D. simulans* were 2,907 bp in length and thus autonomous. All *P* elements shared nucleotide “A” at the position 2,040. Considering that, in the single nucleotide polymorphism (SNP) at 2,040 of *P* elements in *D. melanogaster*, “G” is common and “A” is rare; they concluded that the *P* element of *D. simulans* was horizontally transmitted from *D. melanogaster* and the HT is likely to be a single event.

Hill, Schlötterer, & Betancourt (2016) demonstrated that crosses of *D. simulans* between the dysgenesis‐susceptible (DS) females and dysgenesis‐inducing (DI) males showed gonadal dysgenesis in the F1 progeny, but not in the reciprocal cross and that F1 females of dysgenesis‐resistant (DR) lines have normal ovaries from the crosses with DS females or DI males. Therefore, DS, DI, and DR strains of *D. simulans* are analogous to M, P, and Q strains of *D. melanogaster,* respectively. They implied that the *P* element causes hybrid dysgenesis in *D. simulans* and that its transposition is regulated by PIWI‐interacting RNAs (piRNA), which is the molecular basis of the cytotype for repressing *P* transposition in *D. melanogaster* (Brennecke et al., 2008; Kelleher, 2016; Khurana et al., 2011). More important, Hill et al. (2016) found that the oldest line of *D. simulans* having *P* elements was established in Portugal in 2006 and such lines emerged in Northeast America in 2008 and that *P* elements spread other regions in Africa and Northwest America from 2009 to 2014. Their results suggested that *P* elements invaded *D. simulans* earlier than 2006. This ongoing spreading of *P* elements serves an irreplaceable model for studying the early stage of TE's life cycle.

The aims of this research are to clarify the invasion of *P* elements in *D. simulans* in Asia. A survey of local populations showed that the *D. simulans P* element currently existed in Japanese populations. We also explored the abilities of *P* elements to cause hybrid dysgenesis and determined the full‐length sequence to identify the inserted chromosomal position as a first step to investigate the molecular nature of TEs in the future. Our results support the single event of HT from *D. melanogaster* to *D. simulans* and suggest a nearly simultaneous invasion in Japan, Africa, Europe, and North America.

## 2 MATERIALS AND METHODS

### 2.1 Fly lines

We used 63 isofemale lines of *D. simulans* from 11 localities in Japan collected from 1976 to 2015 (Supporting information Table S1). Three sets of isofemale lines, MSO12, FWU12, and Hikone15, were established with inseminated females collected by banana bait traps. The SPP, OGS, KMM, OEB, AM06, and TSM lines were provided by the Ehime Drosophila Stock Center. TN samples were caught in 2010 and ethanol‐preserved. Rakujuen (RM1) is one of the oldest laboratory stocks of *D. simulans* in Japan, established in 1976 (Kawanishi & Watanabe, 1977). Moreover, two lines of *D. melanogaster*, Harwich and Canton S, were used as P and M strains of the P‐M system of *D. melanogaster,* respectively. Harwich was provided Kyoto Drosophila Stock Center. Flies were maintained on standard food medium at 25°C except for the GD test (see below).

### 2.2 Gonadal dysgenesis tests

According to the standard method for the GD test in the P‐M system in *D. melanogaster* (Engels & Preston, 1980; Kidwell et al., 1977)*,* a set of diagnostic crosses, cross A (DS females × tested males) and cross A* (tested females × DI males), were performed for the Hikone15 lines at 29°C (Hill et al., 2016). All F1 females were individually dissected in 5–7 days after eclosion and the GD score was calculated for each line as the percentage of undeveloped ovaries. We used RM1 and TSM31 as the control of DS and DI strains, respectively. The strain type was defined according to the previous studies; DI strains (more than 10% GD in cross A and less than 10% GD in cross A*), DS strains (less than 10% GD in cross A and more than 10% GD in cross A*), and DR strains (less than 10% GD in both crosses) (Hill et al., 2016; Kidwell, 1993). Similar set of diagnostic crosses were performed for the four Hikone15 lines (15‐5, 15‐10, 5‐14, and 15‐26) at 25°C.

Differences in ratio of dysgenic females between reciprocal cross were tested by Fisher's exact test (FET). Differences in GD% between two temperatures were also tested by FET. The statistical significant levels for multiple comparisons were corrected by the Bonferroni method.

### 2.3 PCR

Genomic DNA was extracted from four adult flies for each isofemale lines, or from one ethanol‐preserved fly, using the GenElute Mammalian Genomic DNA miniprep kit (Sigma‐Aldrich). After ethanol precipitation DNA was dissolved in 30 μl of sterile distilled water. Using the genomic DNA as templates, *P* elements of *D. simulans* were amplified with Ex Taq (Takara) by primer sets as follows: P176F (5′‐CAAAGCTGTGACTGGAGTAA‐3′) and P2812R (5′‐GTCGTATTGAGTCTGAGTGA‐3′), or P357F (5′‐AACGCAGATGCCGTACCTAG‐3′) and P2770R (5′‐AACCCTTAGCATGTCCGTGG‐3′). The PCR reaction conditions for 30 cycles were denaturing at 98°C for 10 s, annealing at 50°C for 30 s and polymerizing at 72°C for 2.5 min for the former set, and denaturing at 98°C for 10 s and annealing at 56°C for 30 s and polymerizing at 72°C for 2.5 min for the latter primer set.

### 2.4 Inverse PCR

Genomic DNA was extracted from ten flies of FWU12‐07 as above and was dissolved in 150 μl of sterile distilled water after ethanol precipitation. We digested the genomic DNA in 20 μl of solution with 15 units of EcoRI (Takara) at 37°C for 1 hr, because the canonical *P* elements should have only one EcoRI site at nucleotide 1711 (Kofler et al., 2015; O'Hare & Rubin, 1983). After heat denaturing EcoRI (65°C, 20 min), digested DNA was self‐ligated with 3 Weiss units of T4 DNA ligase (Promega) at 4°C for overnight. For convenience, hereafter, we call the part of *P* element 5′‐side of the EcoRI site “Left side” (~1.7 kb), and that the 3′‐side of the EcoRI site “Right side” (~1.2 kb). Inverse PCR was performed with the primer sets of Pinv HaeIII‐up (5′‐AAATTCGTCCGCACACAACC‐3′) and Pinv HaeIII‐down (5′‐AATCTTCACGGACACGCAGA‐3′) for the Left side with 0.4 units of Ex Taq (Takara 5 units/μl) in a total volume of 25 μl. PCR conditions were as follows: preheat at 95°C for 3 min, followed by 32 cycles of denaturation at 95°C for 30 s, annealing at 60°C for 30 s and extension at 72°C for 1 min, and additional extension at 72°C for 7 min.

After electrophoresis in a 0.8% agarose gel (Agarose S, Nippon gene), DNA fragments larger than 2.2‐kb were separately extracted and purified using Wizard SV Gel and PCR Clean‐Up System (Promega). The purified DNA was inserted into pGEM vector using pGEM‐T Easy Vector systems (Promega), and transformed to *E. coli* host, JM109. The flanking genomic sequence was determined using the Pinv HaeIII‐up or Pinv HaeIII‐down primer (see above). The obtained sequence was searched for *D. simulans* in FlyBase [http://flybase.org/] using Blast Search. Based on a concatenated sequence of determined and retrieved sequences from the database, a primer pair, 3L2ForNew (5′‐TGATGTGCGTCATTCAGCTT‐3′) and 3L2RevNew (5′‐CGACGAGAGGGAAATGAAAA‐3′), was designed to determine the size and structure of the inserted *P* element using Primer3Plus on the web [http://www.bioinformatics.nl/cgi-bin/primer3plus/primer3plus.cgi/]. The PCR condition was preheat at 95°C for 3 min, followed by for 32 cycles were denaturing at 95°C for 30 s, annealing at 50°C for 30 s and polymerizing at 72°C for 4 min.

### 2.5 Nucleotide sequencing

The DNA fragment was inserted into pGEM vector using pGEM‐T Easy Vector systems (Promega) and BigDye Terminator ver. 3.1 (Thermo Fisher Scientific) and Genetic Analyzer 3100 or 3500 (Thermo Fisher Scientific) were used for DNA sequencing. Cycling conditions were as follows: incubation at 96°C for 1 min followed by 25 cycles of 96°C for 10 s, 50°C for 5 s, and 60°C for 4 min. We used eight primers as follows: P410F (5′‐AGAAGGCTATACCAGTGGGAG‐3′), P759F (5′‐ATTTCCTTTGCCCAGTCGTAC‐3′), P1227F (5′‐ACCTGGTTTAGCCATCCTGC‐3′), P1632F (5′‐GAGTGCTCGCAACCTTATGG‐3′), P1894F (5′‐TCGACCATCCCACTCCACTG‐3), P2250F (5′‐TGAGCCTGTCGATGATGAGC‐3′), M13M4, and M13reverse. The sequence of genomic *P* element obtained in this study was deposited in the DDBJ with the accession number LC274660.

## 3 RESULTS

### 3.1 *P* element sequence in *D. simulans* in Japan

**Figure 2:**
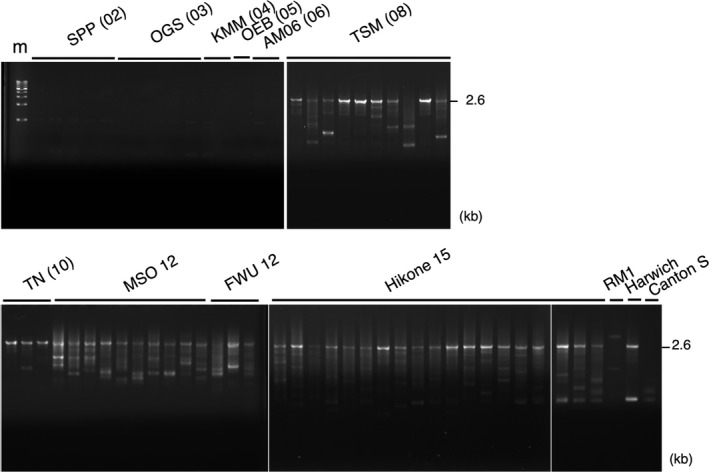
Genomic *P* elements in *Drosophila simulans* in Japan. The primer set of P176F and P2812R was used for PCR. The expected size of the full‐sized *P* elements is indicated as 2.6 kb. The samples were applied from left to right: SPP (02): ‐7, ‐8, ‐28, ‐29, and ‐30; OGS (03): ‐C27, ‐C30, ‐C31, ‐H3, and ‐H4; KMM (04): ‐1 and ‐2; OEB (05); AM06 (06): ‐3 and ‐4; TSM (08): ‐3, ‐27, ‐30, ‐31, ‐32, ‐36, ‐39, ‐58, ‐59, and ‐60; TN (10): ‐134, ‐136, and ‐140; MSO12: ten lines from ‐1 to ‐10; FWU12: ‐4, ‐6, and ‐7; Hikone15: ‐1 to ‐5, ‐7 to ‐11, and ‐13 to ‐21; and RM1 established in 1976. Harwich and Canton S were the standard P and M strains of the P‐M system of *D. melanogaster*. m: 1 kb DNA ladder marker (Takara)

To detect *P* element homologous sequences, we examined genomic DNAs from 63 isofemale lines of *D. simulans* from 11 Japanese local populations established from 1976 to 2015. PCR primer pairs, P176F and P2812R, were designed within the *P* element sequence. Thus, a full‐sized element will generate a product 2.6 kb long. All lines established after 2008 carried copies of *P* sequences containing full‐sized and internally truncated elements (Figure 2). The oldest lines carrying *P* elements were the TSM lines, established in Tsushima Island in 2008. As for the smaller elements, their contents, structure and number, seemed to vary from line to line, and there was no specific small element commonly existing across populations. The 15 lines established before 2006 amplified no *P* element homologous sequence. Similar results were obtained with another set of *P* primers, P257F and P2770R (Supporting information Figure S1). The RM1 showed a few faint signals in Figure 2, but nothing in another PCR (Supporting information Figure S1), suggesting that they were not specific products of *P* sequences.

**Figure 3:**
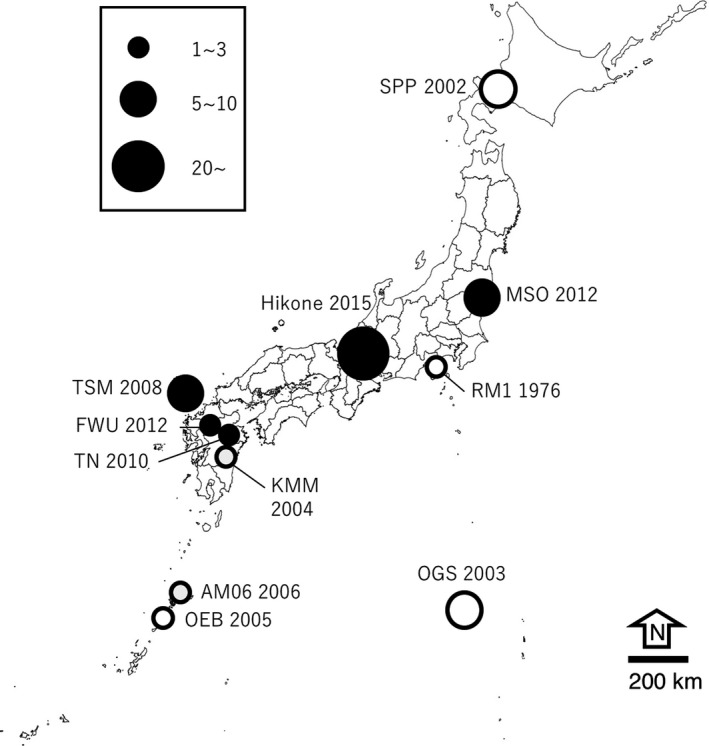
Geographic and chronological distribution of *P* elements in *Drosophila simulans* in Japan. A Closed circle indicates that all lines carried genomic *P* elements, and an open circle indicates no copy of *P* element. The number of lines examined is approximately depicted by the size of each circle. See Supporting information Table S1 and Figure 2 for detail

Recent occurrence of *P* element does not appear limited in any small area, but suggests comparatively wide distribution in the Japanese archipelago. The local populations showed a monotonous result of with or without *P* sequence, hence no polymorphism in each population (Figure 3).

### 3.2 Full‐length *P* element in *D. simulans*

**Figure 4:**
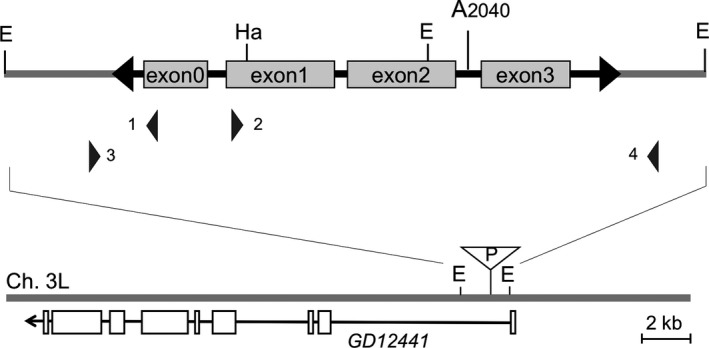
A complete *P* element in *Drosophila simulans* in FWU12‐7. A 2,907‐bp *P* element was located at GCACAGCC at 16,390,349‐16,390,356 of chromosome arm 3 L as the flanking target duplication. The nucleotide sequence of the *P* element was thoroughly identical, containing the A at the position 2,040, to that of the originally invaded element in *D. simulans* (Kofler et al., 2015). Approximate positions of the PCR primers are indicated; 1: Pinv HaeIII‐up, 2: Pinv HaeIII‐down, 3: 3L2RevNew, and 4: 3L2ForNew. The primer set 1&2 was used for inverse PCR and 3&4 for amplifying the region containing the *P* element. This element inserted in the first intron of *GD12441* in the opposite direction. EcoRI sites (E) at 16,389,125 for the left and at 16,391,252 for the right side and HaeIII site (Ha) inside *P* element are indicated in the bottom map

To obtain a complete sequence of the genomic *P* elements and identify the insertion site, we carried out inverse PCR with FWU12‐07, because it appeared to have some full‐sized and only a few other internally truncated smaller *P* elements in the genome (Figure 2, Supporting information Figure S1). We designed primer sets for 14 possible templates containing the flanking sequences of *P* element. We successfully obtained a 3.5‐kb fragment from a template, 3L#2. The nucleotide sequence was determined (3,447 nt). The inserted *P* element was the canonical complete element of 2,907 bp and the nucleotide at position 2,040 was “A”, thus the same as the *P* element that recently identified in *D. simulans* populations (Figure 4). Its upstream 154‐bp and downstream 385‐bp flanking sequences consistently indicated that the element is inserted in an 8 bp of GCACAGCC at 16,390,349–16,390,356 on the chromosome 3 L as the target. The insertion point is in the first intron of *GD12441* gene, which is 19.6 kb in size and deduced to encode a glutamate synthase (FlyBase ID: FBgn0184173). Transcriptional direction of the *P* element was opposite to that of *GD12441*. The EcoRI sites presumed at the both ends in the initial digestion of inverse PCR were confirmed 1,231‐bp upstream and 904‐bp downstream of the target.

In addition, we determined the nucleotide sequence of 17 DNA fragments obtained from MSO12, Hikone15s‐4, and TSM58 by PCR above. The nucleotide was “A” at the position 2,040 in each amplicon, with no exception (Supporting information Figure S2).

### 3.3 Hybrid dysgenesis of the flies in *D. simulans* in Japan

**Figure 5:**
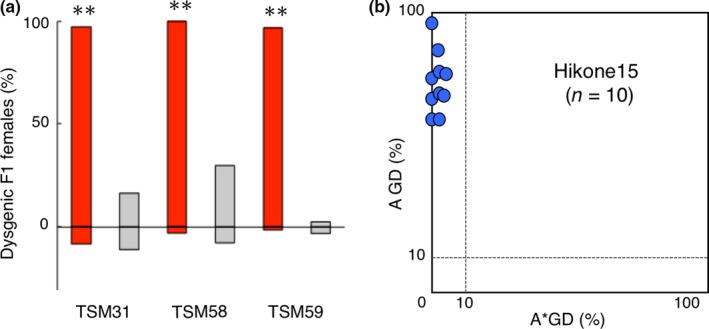
Hybrid dysgenesis in *Drosophila simulans* in Japan. (a) Fraction of dysgenic F1 females for TSM lines in both directions of each set of reciprocal crosses, with RM1 female (positive direction) and with RM1 males (negative direction). Crosses were performed at 29°C (red bar) and 25°C (gray bar). **Difference in the numbers of normal and dysgenic females was significant between the reciprocal crosses (FET,* p *<* *0.01). See Supporting information Table S2 for detail. (b) A–A* graph of Hikone15 lines. Each dot indicates the dysgenic properties of one line. Dots gathered along the vertical axis, implying that they have higher inducibilities (more than 10% of the cross A GD%) and no, if any, susceptibilities (<10% of cross A* GD%), thus DI strains. See Supporting information Table S3 for detail. *n*: the number of lines examined

To know the phenotypic characteristics of the current *D. simulans* in Japan, we carried out the gonadal dysgenesis (GD) test. RM1 was tentatively used as a DS strain because it was free of the *P* element sequence in its genome (Figure 2, Supporting information Figure S1). First, each of three TSM lines was crossed to RM1 in both directions and F1 females were individually dissected in 5–7 days after eclosion to evaluate the development of ovaries. There was a large difference in the fraction of dysgenic females between the offspring in each reciprocal crosses at 29°C (FET, *p *<* *0.01), but not at 25°C (Figure 5a). As a result, unidirectional gonadal dysgenesis was observed in all three lines examined at 29°C. Males of TSM lines induced significantly higher levels (more than 96%) of dysgenic F1 females when crossed to RM1 females. Thus, hereafter we tentatively used TSM31 as a control for the DI strain having both strong inducing ability and high repression potential of hybrid dysgenesis, and RM1 as a control of the DS strain having neither.

A series of GD tests was carried out for the Hikone15 lines at 29°C. GD% of the cross A (RM1 females × tested males) varied from 61.2 to 96.2%, suggesting considerable levels of inducing ability of *P* transposition in each line; GD% of cross A* (tested females x TSM31 males) were less than 4.7%, indicating almost full potential of repression (Figure 5b, Supporting information Table S3). To confirm the effects of high temperature of development in the GD test for *D. simulans*, we again performed GD test for the four lines, Hikone15‐5, 15‐10, 15‐14, and 15‐26, at 25°C. Significant differences were detected in GD% between temperatures, 25°C and 29°C, in the cross A (FET, *p *<* *0.01), but not in the cross A* (Supporting information Table S3 for detail). Therefore, these ten Hikone lines were, from moderate to strong, DI strains. One line, Hikone15‐16, showed prominently strong inducibility, similar to TSM31.

## 4 DISCUSSION

### 4.1 *P* element invasion of *D. simulans* populations in Japan

The previous studies suggested that *P* elements invaded *D. simulans* probably somewhere in Europe, Africa, and North America earlier than 2006, and spread widely in many other areas investigated until 2014 (Hill et al., 2016; Kofler et al., 2015). These observations led us to assume that *P* elements would spread gradually, or from one population to the neighbor step by step, even if it proceeded much more quickly than in *D. melanogaster*. However, based on the present survey of *D. simulans* populations in Japan, *P* element copies were detected in all isofemale lines established after 2008, but no copy before 2006. *P* elements in Japanese populations are likely the descendants of the original *P* element, because they all shared “A” at the nucleotide position 2,040, as in European, African, and American populations. This supports the hypothesis of single event of HT in *D. simulans* (Kofler et al., 2015), and also implies that *P* elements reached to the east end of Asia almost simultaneously to other areas. Therefore, *P* elements dispersed worldwide in *D. simulans* in a curiously short time, and the spreading pattern is not as previously assumed. *P* elements are not likely to spread via a combination of the usual migration of individuals and vertical transmission in *D. simulans*. According to Kofler et al. (2015) human activity of transportation could help flies to move a long distance in a short time. If this is the case, *P* elements must have formed novel colonizing centers at many points of the earth and then dispersed in each area. To understand the dynamics of newly invaded TEs, further investigation of the current local populations for detail distribution of *P* elements is under study, because the numbers and localities of the lines examined in the present study were limited, partly due to the availability of the lines kept in lab and the stock centers.

### 4.2 Dysgenic properties associated with *P* elements in *D. simulans*

Our present GD test showed many strong DI strains, which can induce *P* element transposition, in the TSM and Hikone lines. This may be consistent with a unidirectional high‐temperature‐dependent ovarian dysgenesis in the reciprocal crosses (Figure 5a) and high GD% only in the cross A at 29°C, but not 25°C in the GD test (Supporting information Table S3). The results of PCR demonstrating apparently many copies of full‐sized *P* elements in the genomes also support this assumption. Four strong DI strains, TSM31, TSM58, TSM59, and Hikone15‐16, will be useful as a strong DI strain in the GD test, like Harwich as the strong P strain in *D. melanogaster* (Kidwell, 1979).

On the other hand, our present study found no DR strain in Japan, contrary to the observation in Africa and North America, where DR strains were frequently observed between 2008 and 2012 (Hill et al., 2016). Milder GD in *D. simulans* was hypothesized as one of the possible reasons of more swift spreading of *P* element than that in *D. melanogaster*, because the DI strains from Georgia showed <74% of inducing ability (Hill et al., 2016). Our present results of predominance of strong or moderate DI strains and no DR strain at least in two independent recent populations in Japan would not support this assumption, though. Rather, strong effect in GD and rapid spread may not be always exclusive.

In relation to the *P* element activity, it is noteworthy that one autonomous *P* element was identified in *D. simulans* in Japan and mapped to an 8‐bp on the third chromosome. Kofler et al. (2015) and Hill et al. (2016) showed the presence of *P* sequences in the genomes of *D. simulans*, many of which are full‐length, and mapped some *P* elements. However, no full‐length *P* element of *D. simulans* has yet been determined for its insertion position, because their results were mainly based on next‐generation sequencing (NGS). NGS serves a powerful tool for genomewide investigation by providing many short‐read sequences, but, even if a whole genome sequence is available as a reference, mapping TEs to the genome is not easy. Therefore, the full‐size *P* element was mapped for the first time in wild *D. simulans* in this study. Investigation of moving of individual *P* element is now under study using this element.

### 4.3 Evolutionary aspect of *P* regulatory systems in *Drosophila* hosts

*P* elements were shown to have changed the host species via HT, from *D. willistoni* to *D. melanogaster* in the 20th century (Daniels et al., 1990), and very recently, from *D. melanogaster* to *D. simulans* (Kofler et al., 2015). The host species invaded by TEs needs to develop regulatory systems against selfish jumping of TEs, but the evolutionary process of establishing suppression is not always clear. In *D. melanogaster*, in addition to the major repression by the piRNAs (Brennecke et al., 2008) and the 66 kDa repressor produced by the full‐length element (O'Hare & Rubin, 1983), some internally deleted variants are thought to play important roles in suppressing *P* transposition, for instance, *A12* (Andrews & Gloor, 1995; Gloor et al., 1993), *D50* (Rasmusson, Raymond, & Simmons, 1993), *SP* (Rasmusson et al., 1993), *SR* (Corish, Black, Featherston, Merriam, & Dover, 1996) and *KP* (Black, Jackson, Kidwell, & Dover, 1987; Jackson, Black, & Dover, 1988; Simmons, Grimes, & Czora, 2016) elements, each of which has a specific size. In particular, *KP* elements of *D. melanogaster* increased in number (Andrews & Gloor, 1995; Black et al., 1987) and became predominant together with full‐length elements (FP + KP predominance) worldwide (Itoh & Boussy, 2002; Itoh, Sasai, Inoue, & Watada, 2001; Itoh, Takeuchi, Yamaguchi, Yamamoto, & Boussy, 2007; Itoh, Woodruff, Leone, & Boussy, 1999; Itoh et al., 2004; Ogura, Woodruff, Itoh, & Boussy, 2007). FP + KP predominance may support an advantageous effect of *KP* elements by preventing *P* elements from transposing. An alternative plausible hypothesis was suggested that *KP* elements have more ability of transposition than other *P* elements (Fukui, Inoue, Yamaguchi, & Itoh, 2008). Based on our PCR analyses in *D. simulans*, all lines examined carried some copies of full‐length and smaller derivative elements. However, in striking contrast to *D. melanogaster*, no specific smaller elements were frequently observed (Figure 1, Supporting information Figure S1).

Only several years may be too short for a unique repressor variant, like *KP* elements, to evolve de novo in *D. simulans*. Such smaller repressor elements, however, were not found in *D. willistoni* (Regner, Pereira, Alonso, Abdelhay, & Valente, 1996), although *D. willistoni* should have the longest evolutionary history with *P* elements among the three species (Powell & Gleason, 1996). On the other hand, repression by the piRNAs was shown to work in *D. simulans* (Hill et al., 2016) and *D. melanogaster* (Brennecke et al., 2008; Khurana et al., 2011), but still unknown in *D. willistoni*. From the view of evolution of repression systems, careful investigation is required, for instance, comparing expression, fine structure, and insertion sites of the genomic *P* elements both between populations and between species.

## CONFLICT OF INTEREST

None declared.

## AUTHOR CONTRIBUTIONS

MI conceived and designed the study. MI, YY, and NI collected some wild flies and established the isofemale lines. YY, NI, MS, and YK contributed to data acquisition and analysis. MI, YY, and NI drafted the first manuscript. MI, NI, YK, YY, and MS contributed to the revision. MI, NI, YK, YY, and MS approved final submission.

## DATA ACCESSIBILITY

DNA sequences DDBJ accession number: LC274660.

## References

[ece34239-bib-0001] Andrews, J. D. , & Gloor, G. B. (1995). A role for the KP leucine zipper in regulating P element transposition in *Drosophila melanogaster* . Genetics, 141, 587–94. 8647395 10.1093/genetics/141.2.587 PMC1206758

[ece34239-bib-0002] Anxolabéhère, D. , Kidwell, M. G. , & Periquet, G. (1988). Molecular characteristics of diverse populations are consistent with the hypothesis of a recent invasion of *Drosophila melanogaster* by mobile *P* elements. Molecular Biology and Evolution, 5, 252–69. 2838720 10.1093/oxfordjournals.molbev.a040491

[ece34239-bib-0003] Anxolabéhère, D. , Nouaud, D. , Periquet, G. , & Tchen, P. (1985). P‐element distribution in Eurasian populations of *Drosophila melanogaster*: A genetic and molecular analysis. Proceedings of the National Academy of Sciences of the United States of America, 82, 5418–22. 10.1073/pnas.82.16.5418 16593591 PMC390580

[ece34239-bib-0004] Ashburner, M. , Golic, K. G. , & Hawley, R. (2005). Drosophila: A laboratory handbook. Cold Spring Harbor, NY: Cold Spring Harbor Laboratory Press, xxviii.

[ece34239-bib-0005] Bergman, C. M. , Quesneville, H. , Anxolabéhère, D. , & Ashburner, M. (2006). Recurrent insertion and duplication generate networks of transposable element sequences in the *Drosophila melanogaster* genome. Genome Biology, 7, R112 10.1186/gb-2006-7-11-r112 17134480 PMC1794594

[ece34239-bib-0006] Biemont, C. , & Vieira, C. (2006). Genetics: junk DNA as an evolutionary force. Nature, 443, 521–4. 10.1038/443521a 17024082

[ece34239-bib-0007] Bingham, P. M. , Kidwell, M. G. , & Rubin, G. M. (1982). The molecular basis of PM hybrid dysgenesis: The role of the P element, a P‐strain‐specific transposon family. Cell, 29, 995–1004. 10.1016/0092-8674(82)90463-9 6295641

[ece34239-bib-0008] Black, D. M. , Jackson, M. S. , Kidwell, M. G. , & Dover, G. A. (1987). KP elements repress P‐induced hybrid dysgenesis in *Drosophila melanogaster* . EMBO Journal, 6, 4125–35. 2832152 10.1002/j.1460-2075.1987.tb02758.x PMC553895

[ece34239-bib-0009] Bonnivard, E. , & Higuet, D. (1999). Stability of European natural populations of *Drosophila melanogaster* with regard to the PM system: A buffer zone made up of Q populations. Journal of Evolutionary Biology, 12, 633–647. 10.1046/j.1420-9101.1999.00063.x

[ece34239-bib-0010] Brennecke, J. , Malone, C. D. , Aravin, A. A. , Sachidanandam, R. , Stark, A. , & Hannon, G. J. (2008). An epigenetic role for maternally inherited piRNAs in transposon silencing. Science, 322, 1387–1392. 10.1126/science.1165171 19039138 PMC2805124

[ece34239-bib-0011] Brookfield, J. F. (1991). Models of repression of transposition in P‐M hybrid dysgenesis by P cytotype and by zygotically encoded repressor proteins. Genetics, 128, 471–86. 1649073 10.1093/genetics/128.2.471 PMC1204483

[ece34239-bib-0012] Capy, P. , Gibert, P. , & Boussy, I. A. (2004). Drosophila melanogaster, Drosophila simulans: So similar, so different. Dordrecht, the Netherlands: Springer 10.1007/978-94-007-0965-2 15088643

[ece34239-bib-0013] Corish, P. , Black, D. M. , Featherston, D. W. , Merriam, J. , & Dover, G. A. (1996). Natural repressors of P‐induced hybrid dysgenesis in *Drosophila melanogaster*: A model for repressor evolution. Genetical Research, 67, 109–21. 10.1017/S0016672300033577 8801184

[ece34239-bib-0014] Craig, N. L. , Craigie, R. , Gellert, M. , & Lambowitz, A. M. (2002). Mobile DNA II. Washington, DC: American Society for Microbiology Press 10.1128/9781555817954

[ece34239-bib-0015] Daniels, S. B. , Chovnick, A. , & Kidwell, M. G. (1989). Hybrid dysgenesis in *Drosophila simulans* lines transformed with autonomous P elements. Genetics, 121, 281–91. 2731724 10.1093/genetics/121.2.281 PMC1203618

[ece34239-bib-0016] Daniels, S. B. , Peterson, K. R. , Strausbaugh, L. D. , Kidwell, M. G. , & Chovnick, A. (1990). Evidence for horizontal transmission of the P transposable element between *Drosophila* species. Genetics, 124, 339–355. 2155157 10.1093/genetics/124.2.339 PMC1203926

[ece34239-bib-0017] Daniels, S. B. , Strausbaugh, L. D. , & Armstrong, R. A. (1985). Molecular analysis of P element behavior in *Drosophila* simulans transformants. Molecular and General Genetics, 200, 258–65. 10.1007/BF00425433 2993820

[ece34239-bib-0018] Drezen, J. M. , Gauthier, J. , Josse, T. , Bezier, A. , Herniou, E. , & Huguet, E. (2017). Foreign DNA acquisition by invertebrate genomes. Journal of Invertebrate Pathology, 147, 157–168. 10.1016/j.jip.2016.09.004 27642089

[ece34239-bib-0019] Engels, W. R. , & Preston, C. R. (1980). Components of hybrid dysgenesis in a wild population of *Drosophila melanogaster* . Genetics, 95, 111–28. 6776005 10.1093/genetics/95.1.111 PMC1214210

[ece34239-bib-0020] Feschotte, C. , & Pritham, E. J. (2007). DNA transposons and the evolution of eukaryotic genomes. Annual Review of Genetics, 41, 331–68. 10.1146/annurev.genet.40.110405.090448 PMC2167627 18076328

[ece34239-bib-0021] Fukui, T. , Inoue, Y. , Yamaguchi, M. , & Itoh, M. (2008). Genomic P elements content of a wild M’ strain of *Drosophila melanogaster*: KP elements do not always function as type II repressor elements. Genes & Genetic Systems, 83, 67–75. 10.1266/ggs.83.67 18379135

[ece34239-bib-0022] Gloor, G. B. , Preston, C. R. , Johnson‐Schlitz, D. M. , Nassif, N. A. , Phillis, R. W. , Benz, W. K. , … Engels, W. R. (1993). Type I repressors of P element mobility. Genetics, 135, 81–95. 8224830 10.1093/genetics/135.1.81 PMC1205629

[ece34239-bib-0023] Higuet, D. , Merçot, H. , Allouis, S. , & Montchamp‐Moreau, C. (1996). The relationship between structural variation and dysgenic properties of P elements in long‐established P‐transformed lines of *Drosophila simulans* . Heredity (Edinb), 77(Pt 1), 9–15. 10.1038/hdy.1996.102 8682694

[ece34239-bib-0024] Hill, T. , Schlötterer, C. , & Betancourt, A. J. (2016). Hybrid dysgenesis in *Drosophila simulans* associated with a rapid invasion of the P‐Element. PLoS Genetics, 12, e1005920 10.1371/journal.pgen.1005920 26982327 PMC4794157

[ece34239-bib-0025] Houck, M. A. , Clark, J. B. , Peterson, K. R. , & Kidwell, M. G. (1991). Possible horizontal transfer of *Drosophila* genes by the mite Proctolaelaps regalis. Science, 253, 1125–8. 10.1126/science.1653453 1653453

[ece34239-bib-0026] Hua‐Van, A. , Le Rouzic, A. , Boutin, T. S. , Filee, J. , & Capy, P. (2011). The struggle for life of the genome's selfish architects. Biology Direct, 6, 19 10.1186/1745-6150-6-19 21414203 PMC3072357

[ece34239-bib-0027] Itoh, M. , & Boussy, I. A. (2002). Full‐size P and KP elements predominate in wild *Drosophila melanogaster* . Genes & Genetic Systems, 77, 259–267. 10.1266/ggs.77.259 12419898

[ece34239-bib-0028] Itoh, M. , Fukui, T. , Kitamura, M. , Uenoyama, T. , Watada, M. , & Yamaguchi, M. (2004). Phenotypic stability of the P‐M system in wild populations of *Drosophila melanogaster* . Genes & Genetic Systems, 79, 9–18. 10.1266/ggs.79.9 15056932

[ece34239-bib-0029] Itoh, M. , Sasai, N. , Inoue, Y. , & Watada, M. (2001). P elements and PM characteristics in natural populations of *Drosophila melanogaster* in the southernmost islands of Japan and in Taiwan. Heredity, 86, 206–212. 10.1046/j.1365-2540.2001.00817.x 11380666

[ece34239-bib-0030] Itoh, M. , Takeuchi, N. , Yamaguchi, M. , Yamamoto, M. T. , & Boussy, I. A. (2007). Prevalence of full‐size P and KP elements in North American populations of *Drosophila melanogaster* . Genetica, 131, 21–8. 10.1007/s10709-006-9109-2 17318316

[ece34239-bib-0031] Itoh, M. , Woodruff, R. C. , Leone, M. A. , & Boussy, I. A. (1999). Genomic P elements and P‐M characteristics of eastern Australian populations of *Drosophila melanogaster* . Genetica, 106, 231–245. 10.1023/A:1003918417012 10897797

[ece34239-bib-0032] Jackson, M. S. , Black, D. M. , & Dover, G. A. (1988). Amplification of KP elements associated with the repression of hybrid dysgenesis in *Drosophila melanogaster* . Genetics, 120, 1003–13. 2852140 10.1093/genetics/120.4.1003 PMC1203564

[ece34239-bib-0033] Kawanishi, M. , & Watanabe, T. (1977). Ecological factors controlling the coexistence of the sibling species *Drosophila simulans* and *D. melanogaster* . Japanese Journal of Ecology, 27, 279–283.

[ece34239-bib-0034] Kelleher, E. S. (2016). Reexamining the P‐Element Invasion of *Drosophila melanogaster* Through the Lens of piRNA Silencing. Genetics, 203, 1513–31. 10.1534/genetics.115.184119 27516614 PMC4981261

[ece34239-bib-0035] Khurana, J. S. , Wang, J. , Xu, J. , Koppetsch, B. S. , Thomson, T. C. , Nowosielska, A. , … Theurkauf, W. E. (2011). Adaptation to P element transposon invasion in *Drosophila melanogaster* . Cell, 147, 1551–63. 10.1016/j.cell.2011.11.042 22196730 PMC3246748

[ece34239-bib-0036] Kidwell, M. G. (1979). Hybrid dysgenesis in *Drosophila melanogaster*: The relationship between the P– M and I– R interaction systems. Genetical Research, 33, 205 10.1017/S0016672300018358

[ece34239-bib-0037] Kidwell, M. G. (1993). Lateral transfer in natural populations of eukaryotes. Annual Review of Genetics, 27, 235–56. 10.1146/annurev.ge.27.120193.001315 8122903

[ece34239-bib-0038] Kidwell, M. G. (1994). The Wilhelmine E. Key 1991 Invitational Lecture. The evolutionary history of the P family of transposable elements. Journal of Heredity, 85, 339–46. 10.1093/oxfordjournals.jhered.a111478 7963451

[ece34239-bib-0039] Kidwell, M. G. , Kidwell, J. F. , & Sved, J. A. (1977). Hybrid Dysgenesis in *Drosophila melanogaster*: A syndrome of aberrant traits including mutation, sterility and male recombination. Genetics, 86, 813–33. 17248751 10.1093/genetics/86.4.813 PMC1213713

[ece34239-bib-0040] Kidwell, M. G. , & Lisch, D. (1997). Transposable elements as sources of variation in animals and plants. Proceedings of the National Academy of Sciences of the United States of America, 94, 7704–7711. 10.1073/pnas.94.15.7704 9223252 PMC33680

[ece34239-bib-0041] Kimura, K. , & Kidwell, M. G. (1994). Differences in P element population dynamics between the sibling species *Drosophila melanogaster* and *Drosophila simulans* . Genetical Research, 63, 27–38. 10.1017/S0016672300032055 8206365

[ece34239-bib-0042] Kofler, R. , Hill, T. , Nolte, V. , Betancourt, A. J. , & Schlötterer, C. (2015). The recent invasion of natural *Drosophila simulans* populations by the P‐element. Proceedings of the National Academy of Sciences of the United States of America, 112, 6659–63. 10.1073/pnas.1500758112 25964349 PMC4450375

[ece34239-bib-0043] Le Rouzic, A. , & Capy, P. (2005). The first steps of transposable elements invasion. Genetics, 169, 1033–1043. 10.1534/genetics.104.031211 15731520 PMC1449084

[ece34239-bib-0044] Le Rouzic, A. , & Capy, P. (2009). Theoretical approaches to the dynamics of transposable elements in genomes, populations, and species. In: Lankenau D.‐H. & Volff J.‐N. (Ed.), Transposons and the Dynamic Genome, (pp. 1‐19). Springer‐Verlag: Berlin, Heidelberg ISBN: 978‐3‐642‐02004‐9.

[ece34239-bib-0045] Lee, Y. C. G. , & Langley, C. H. (2012). Long‐term and short‐term evolutionary impacts of transposable elements on *Drosophila* . Genetics, 192, 1411–1432. 10.1534/genetics.112.145714 22997235 PMC3512147

[ece34239-bib-0046] Lewis, S. H. , Quarles, K. A. , Yang, Y. , Tanguy, M. , Fr Zal, L. , Smith, S. A. , … Giraud, I. (2018). Pan‐arthropod analysis reveals somatic piRNAs as an ancestral defence against transposable elements. Nature Ecology & Evolution, 2, 174 10.1038/s41559-017-0403-4 29203920 PMC5732027

[ece34239-bib-0047] Montchamp‐Moreau, C. (1990). Dynamics of P‐M hybrid dysgenesis in P‐transformed lines of *Drosophila simulans* . Evolution, 44, 194–203. 28568199 10.1111/j.1558-5646.1990.tb04289.x

[ece34239-bib-0048] Ogura, K. , Woodruff, R. C. , Itoh, M. , & Boussy, I. A. (2007). Long‐term patterns of genomic P element content and P‐M characteristics of *Drosophila melanogaster* in eastern Australia. Genes & Genetic Systems, 82, 479–87. 10.1266/ggs.82.479 18270438

[ece34239-bib-0049] O'Hare, K. , & Rubin, G. M. (1983). Structures of P transposable elements and their sites of insertion and excision in the *Drosophila melanogaster* genome. Cell, 34, 25–35. 10.1016/0092-8674(83)90133-2 6309410

[ece34239-bib-0050] Orgel, L. E. , & Crick, F. H. C. (1980). Selfish DNA: The ultimate parasite. Nature, 284, 604–607. 10.1038/284604a0 7366731

[ece34239-bib-0051] Pinsker, W. , Haring, E. , Hagemann, S. , & Miller, W. J. (2001). The evolutionary life history of P transposons: From horizontal invaders to domesticated neogenes. Chromosoma, 110, 148–158. 10.1007/s004120100144 11513290

[ece34239-bib-0052] Powell, J. R. , & Gleason, J. M. (1996). Codon usage and the origin of P elements. Molecular Biology and Evolution, 13, 278–279. 10.1093/oxfordjournals.molbev.a025564 8583900

[ece34239-bib-0053] Rasmusson, K. E. , Raymond, J. D. , & Simmons, M. J. (1993). Repression of hybrid dysgenesis in *Drosophila melanogaster* by individual naturally occurring P elements. Genetics, 133, 605–22. 8384145 10.1093/genetics/133.3.605 PMC1205347

[ece34239-bib-0054] Regner, L. P. , Pereira, M. S. , Alonso, C. E. , Abdelhay, E. , & Valente, V. L. (1996). Genomic distribution of P elements in *Drosophila willistoni* and a search for their relationship with chromosomal inversions. Journal of Heredity, 87, 191–8. 10.1093/oxfordjournals.jhered.a022984 8683096

[ece34239-bib-0055] Scavarda, N. J. , & Hartl, D. L. (1984). Interspecific DNA transformation in *Drosophila* . Proceedings of the National Academy of Sciences of the United States of America, 81, 7515–7519. 10.1073/pnas.81.23.7515 6095302 PMC392177

[ece34239-bib-0056] Scavarda, N. J. , & Hartl, D. L. (1987). Germ line abnormalities in *Drosophila simulans* transfected with the transposable P element. Journal of Genetics, 66, 1–15.

[ece34239-bib-0057] Schaack, S. , Gilbert, C. , & Feschotte, C. (2010). Promiscuous DNA: Horizontal transfer of transposable elements and why it matters for eukaryotic evolution. Trends in Ecology & Evolution, 25, 537–546. 10.1016/j.tree.2010.06.001 20591532 PMC2940939

[ece34239-bib-0058] Senti, K.‐A. , Jurczak, D. , Sachidanandam, R. , & Brennecke, J. (2015). piRNA‐guided slicing of transposon transcripts enforces their transcriptional silencing via specifying the nuclear piRNA repertoire. Genes & Development, 29, 1747–1762. 10.1101/gad.267252.115 26302790 PMC4561483

[ece34239-bib-0059] Simmons, M. J. , Grimes, C. D. , & Czora, C. S. (2016). Cytotype regulation facilitates repression of hybrid dysgenesis by naturally occurring KP elements in *Drosophila melanogaster* . G3 (Bethesda), 6, 1891–1897. 10.1534/g3.116.028597 27172198 PMC4938643

[ece34239-bib-0060] Venner, S. , Miele, V. , Terzian, C. , Biemont, C. , Daubin, V. , Feschotte, C. , & Pontier, D. (2017). Ecological networks to unravel the routes to horizontal transposon transfers. PLoS Biology, 15, e2001536 10.1371/journal.pbio.2001536 28199335 PMC5331948

