# Supplementary figures and images for: The P element invaded rapidly and caused hybrid dysgenesis in natural populations of Drosophila simulans in Japan
Source: Ecol Evol. 2018 Sep 4;8(19):9590–9. doi: 10.1002/ece3.4239 (PMC6202753; doi:10.1002/ece3.4239)

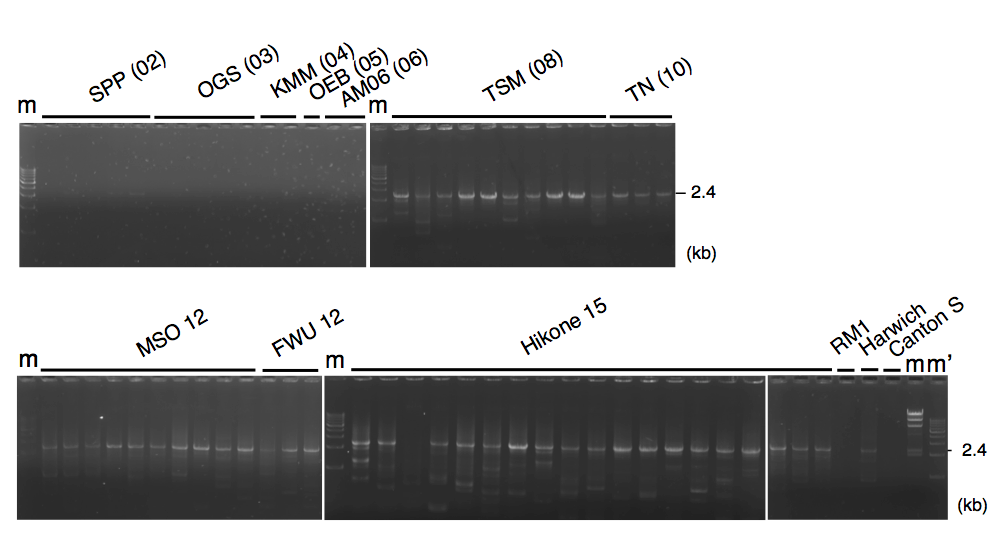

Supplement: Supplementary file 1 [file ECE3-8-9590-s001.tiff]

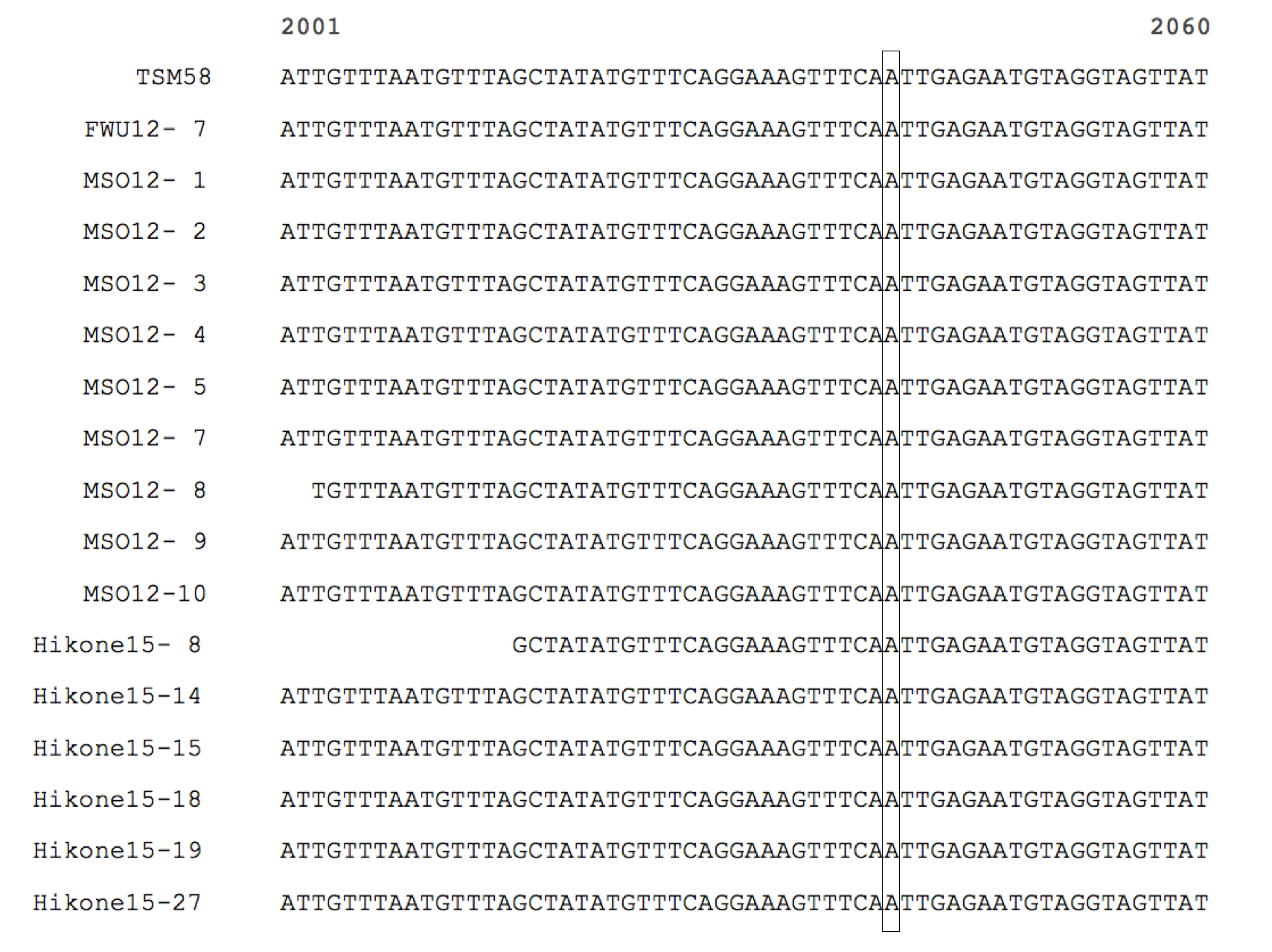

Supplement: Supplementary file 2 [file ECE3-8-9590-s002.tiff]
